# Does Obesity Influence the Outcomes of Supine Mini-Percutaneous Nephrolithotomy? A Retrospective Observational Study

**DOI:** 10.3390/medicina62101950

**Published:** 2026-10-09

**Authors:** Zoltán Kiss, Tibor Flaskó, Attila Nagy, Ioannis Kartalas Goumas, Mihály Murányi

**Affiliations:** 1 Department of Urology, University of Debrecen, 4032 Debrecen, Hungary; flash@med.unideb.hu (T.F.); muranyi.mihaly@med.unideb.hu (M.M.); 2 Department of Health Informatics, Faculty of Health Sciences, University of Debrecen, 4032 Debrecen, Hungary; nagy.attila@sph.unideb.hu; 3 Clinical Institute Beato Matteo, 27029 Vigevano, Italy; ikg.encroach194@passmail.net

**Keywords:** supine mini-percutaneous nephrolithotomy, obesity, kidney stone, postoperative outcomes

## Abstract

*Background and Objectives:* The objective of this study is to evaluate the impact of obesity on postoperative outcomes following supine mini-percutaneous nephrolithotomy (mini-PCNL). *Materials and Methods:* This retrospective observational study included 110 consecutive patients who underwent supine mini-PCNL between October 2024 and March 2026. Patients were stratified into non-obese (BMI < 30 kg/m^2^, *n* = 59) and obese (BMI ≥ 30 kg/m^2^, *n* = 51) groups. Preoperative characteristics, stone-related parameters, and perioperative outcomes were compared. The primary endpoint was the 30-day postoperative complication rate according to the modified Clavien–Dindo classification. Secondary outcomes included operative time, hemoglobin drop, stone-free rate (SFR), and postoperative day 1 discharge rate. *Results:* Obese patients presented with greater cumulative stone diameter and skin-to-stone distance. Operative time was longer in the obese group (45 [40–60] min vs. 35 [30–60] min; *p* = 0.029). However, after adjustment for cumulative stone diameter and age, obesity was not independently associated with operative time, whereas stone size remained a significant predictor. No significant differences were observed between groups regarding hemoglobin drop (obese cohort: 9.27 ± 8.22 g/L vs. non-obese cohort: 8.88 ± 7.35 g/L; *p* = 0.95), SFR (obese cohort: 94.1% vs. non-obese cohort: 89.8%; *p* = 0.41), postoperative day 1 discharge (obese cohort: 86.3% vs. non-obese cohort: 89.8%; *p* = 0.768), or overall complication rates (obese cohort: 5.88% vs. non-obese cohort: 6.78%; absolute risk difference, −0.9 percentage points; 95% CI, −10.0 to 8.2; *p* = 1.000). *Conclusions:* In this cohort, no statistically significant differences were observed between obese and non-obese patients in the assessed perioperative outcomes, except for operative time, which was longer in obese patients, and the observed difference appeared to be largely explained by differences in stone burden in the present cohort. These findings support the feasibility of standardized supine mini-PCNL in obese patients, although larger prospective studies are needed to confirm these results.

## 1. Introduction

The prevalence of obesity is increasing worldwide, likely due to reduced energy expenditure and sedentary lifestyles [1]. Obesity is associated with an increased risk of kidney stone formation and high recurrence rates [2]. Percutaneous nephrolithotomy (PCNL) remains an effective treatment option in obese patients with kidney stones; however, it poses substantial technical and anesthetic challenges due to patient- and comorbidity-related factors. The reported efficacy and safety of PCNL in obese populations remain controversial across studies. Notably, most available data is derived from standard PCNL performed using 24–30 Ch Amplatz sheaths, predominantly in the prone position [3,4,5,6,7]. Although some recent studies have evaluated supine mini-PCNL in obese patients, evidence regarding the outcomes of standardized supine mini-PCNL in this population remains limited [8,9]. Specifically, the relative contribution of obesity itself, as opposed to obesity-associated stone burden, to perioperative outcomes has been insufficiently explored. Therefore, the aim of this retrospective study was to evaluate the safety and efficacy of supine mini-PCNL in obese patients.

## 2. Materials and Methods

### 2.1. Study Design

**Figure 1:**
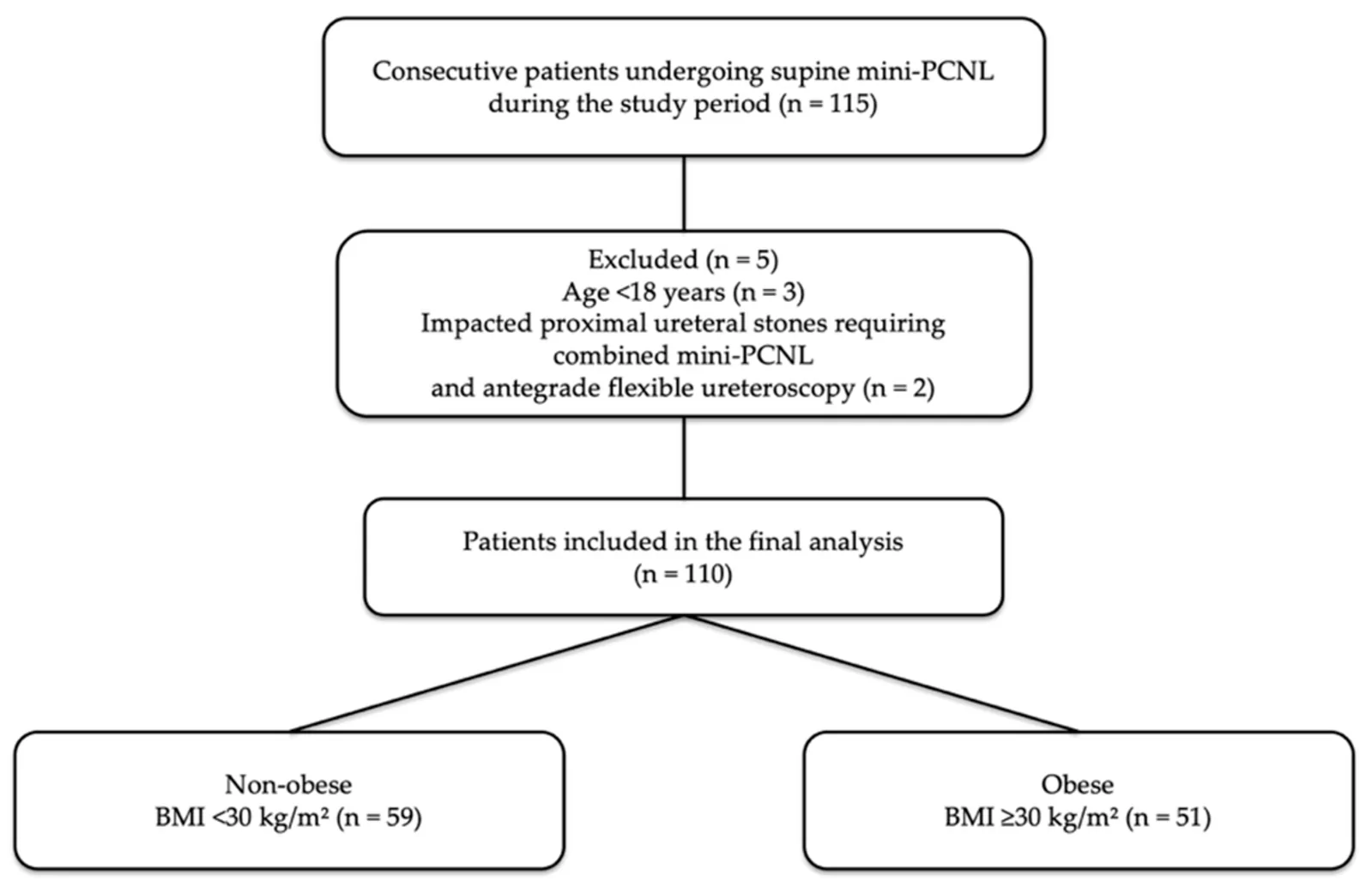
Flow diagram of patient selection and study group allocation.

Between October 2024 and March 2026, 110 consecutive patients underwent supine non-suction mini-PCNL at a tertiary referral center. Following approval from the Regional Institutional Research Ethics Committee (IRB No. 7492-2026 RKEB) and acquisition of written informed consent, data were retrospectively collected. The study was conducted in accordance with the principles of the Declaration of Helsinki. All personally identifiable patient information was removed after data collection to protect patient privacy. Patients were categorized into two groups based on the body mass index (BMI) cut-off of 30 kg/m^2^—non-obese (BMI < 30 kg/m^2^) and obese (BMI ≥ 30 kg/m^2^)—according to the World Health Organization definition of obesity [10]. Inclusion criteria were age >18 years and the presence of single or multiple renal stones. Exclusion criteria included bleeding disorders, pregnancy, untreated urinary tract infections (UTIs), and isolated ureteral stones. The patient selection process is summarized in Figure 1. All procedures were performed by a single surgeon experienced in PCNL who had surpassed the learning curve for mini-PCNL prior to study initiation.

**Figure 2:**
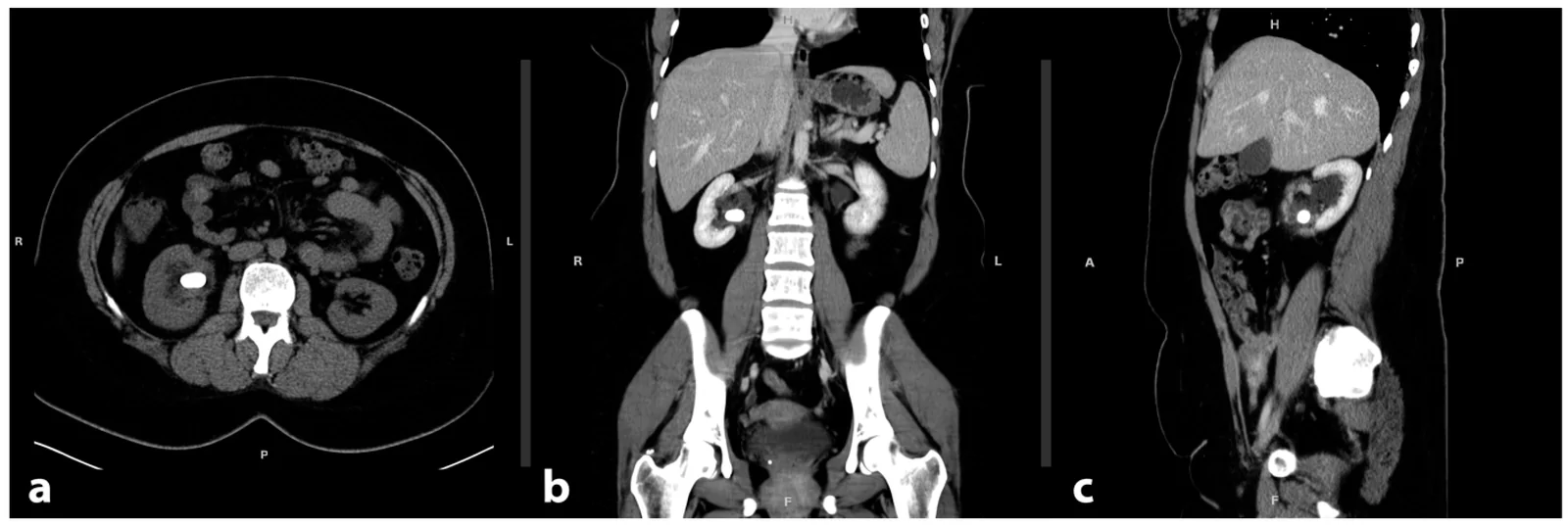
Representative axial (**a**), coronal (**b**), and sagittal (**c**) CT images of a patient with a BMI of 44 kg/m^2^.

Patient demographics, including age, sex, BMI and American Society of Anesthesiologists (ASA) score, were analyzed. Baseline comorbidities, including hypertension and diabetes mellitus, were also recorded. All patients underwent low-dose abdominal and pelvic computed tomography (CT) from which the maximum diameter and the density of the stones was evaluated (Figure 2). In patients with multiple stones, individual stone diameters were summed to calculate cumulative stone size. Skin-to-stone distance (SSD) was also evaluated using preoperative CT imaging. Surgical complexity was assessed using the Guy’s stone score. Midstream urine cultures and laboratory parameters (creatinine, hemoglobin) were also analyzed.

### 2.2. Surgical Technique

The surgical technique and patient positioning have been described previously in our report on supine PCNL [11]. In the current study, the same positioning principles were used; however, a mini-PCNL approach was applied. Briefly, under general anesthesia, patients were positioned in the Barts “flank-free” modified supine position with a 15-degree tilt, ensuring appropriate padding and protection of pressure points. In obese patients, adhesive tapes were used to retract the abdominal wall towards the contralateral side, thereby improving access to the operative field. Following ultrasound-guided renal puncture, tract dilatation was performed under fluoroscopic guidance using the Storz MIP M one-step dilator (Karl Storz, Tuttlingen, Germany), followed by the placement of a 16.5–17.5 Ch, 18-cm-long Amplatz sheath and a 12 Ch nephroscope. For lithotripsy, a 30 W holmium laser (Quanta System S.p.A., Samarate, Italy) with a 550-µm laser fiber was used at a maximum power up to 20 W. The stone fragments were evacuated using the Venturi effect. At the end of the procedure, a 26 or 28 cm long, 6 Ch DJ stent (Urotech GmbH, Achenmühle, Germany) was placed in a retrograde fashion. No nephrostomy tube was placed.

### 2.3. Outcome Measures

The primary outcome of the study was the 30-day complication rate, assessed according to the modified Clavien–Dindo classification system [12,13]. Secondary outcomes included operative time, hemoglobin drop, stone-free rate (SFR), and postoperative day 1 discharge rate. Operative time and length of hospital stay were documented for all patients. Hemoglobin and creatinine levels were measured on the first postoperative day. SFR was evaluated 1 month after the procedure using kidney–ureter–bladder (KUB) radiography and ultrasonography. As non-contrast CT was not routinely performed during follow-up, residual fragments <4 mm were considered indicative of stone-free status. This definition differs from contemporary European Association of Urology guideline recommendations, which apply more stringent imaging criteria and smaller residual fragment thresholds.

### 2.4. Statistical Analysis

The distribution of continuous variables was evaluated using the Shapiro–Wilk test. Normally distributed variables were reported as mean ± standard deviation, while non-normally distributed variables were reported as median with interquartile range. Group comparisons were performed using the independent samples *t*-test for normally distributed continuous variables and the Mann–Whitney U test for non-normally distributed variables. Categorical variables were expressed as numbers and percentages and were compared using Pearson’s chi-squared test or Fisher’s exact test, as appropriate. For sparse categorical data and individual category comparisons, Fisher’s exact test was used. Multivariable linear regression analyses with robust standard errors were performed to identify independent predictors of continuous outcome variables and to adjust for potential confounders. As a post hoc sensitivity analysis, an expanded multivariable linear regression model was also performed including obesity, cumulative stone diameter, age, stone density, Guy’s stone score, number of stones, diabetes mellitus, and ASA status. As an additional sensitivity analysis, BMI was evaluated as a continuous variable rather than dichotomized according to the obesity threshold. Covariates were selected based on clinical relevance rather than statistical significance alone. Because SSD is closely related to obesity and may partly represent a mediator of the technical effects of obesity rather than a conventional confounder, SSD was evaluated separately in a sensitivity analysis. Multicollinearity was assessed using variance inflation factors (VIFs). Given the limited number of outcome events, post hoc exploratory parsimonious logistic regression models including obesity and cumulative stone diameter were used to evaluate associations with 30-day complications and failure to achieve stone-free status. Missing data were assessed for all variables included in the analyses; no missing values were identified in the final study cohort. A *p*-value < 0.05 was considered statistically significant. The 30-day complication rate was predefined as the primary endpoint of the study. Analyses of secondary outcomes were considered exploratory and hypothesis-generating; therefore, no formal adjustment for multiple comparisons was applied. The multivariable and sensitivity analyses were performed post hoc and should therefore be considered exploratory. Statistical analyses were performed using Intercooled Stata v18.0. (StataCorp. 2023. Stata Statistical Software: Release 18. StataCorp LLC., College Station, TX, USA).

## 3. Results

Demographic and clinical characteristics of the study population are presented in Table 1.

Of the 110 patients, 59 (53.64%) were assigned to the non-obese group and 51 (46.36%) to the obese group. Among the 51 obese patients, 25 (49.0%) had a BMI of 30.0–34.9 kg/m^2^, 20 (39.2%) had a BMI of 35.0–39.9 kg/m^2^, and 6 (11.8%) had a BMI ≥ 40 kg/m^2^. The distribution of ASA scores demonstrated a borderline significant difference between the groups, with a higher proportion of ASA II and III patients in the obese cohort. This is in line with the significantly higher prevalence of hypertension and diabetes mellitus among obese patients. Regarding stone characteristics, cumulative stone diameter and SSD were significantly greater in the obese group, whereas stone density was significantly higher in the non-obese cohort. Median operative time was significantly longer in the obese group compared to non-obese patients (45 [40–60] min versus 35 [30–60] min, respectively: *p* = 0.029). However, obese patients also had significantly greater cumulative stone diameter. After adjustment for cumulative stone diameter and age, obesity was no longer independently associated with operative time (β = 1.81 min, 95% CI: −5.99 to 9.61; *p* = 0.647), whereas cumulative stone diameter remained a significant predictor of longer operative time (β = 0.74 min/mm, 95% CI: 0.49 to 0.99; *p* < 0.001). In an additional sensitivity analysis treating BMI as a continuous variable, BMI was not independently associated with operative time after adjustment for cumulative stone diameter and age (β = 0.03 min per kg/m^2^; 95% CI, −0.72 to 0.77; *p* = 0.944). In an expanded post hoc sensitivity model including obesity, cumulative stone diameter, age, stone density, Guy’s stone score, number of stones, diabetes mellitus, and ASA status, obesity remained non-significantly associated with operative time (β = 0.67 min; *p* = 0.864), whereas cumulative stone diameter remained significantly associated with longer operative time (β = 0.84 min/mm; *p* < 0.001). In a separate sensitivity analysis including SSD, obesity remained non-significantly associated with operative time (β = 6.68 min; *p* = 0.129), whereas cumulative stone diameter remained significantly associated with longer operative time (β = 0.73 min/mm; *p* < 0.001). No relevant multicollinearity was observed (all VIFs ≤ 2.12). All procedures were completed in a single session, and no staged procedures were required in either group.

Mean preoperative hemoglobin levels were significantly higher in the obese group. Similarly, postoperative hemoglobin values remained higher in obese patients (135.3 ± 18.7 g/L vs. 128.1 ± 16.0 g/L; *p* = 0.031). However, the mean decrease in hemoglobin levels did not differ significantly between obese and non-obese patients (9.27 ± 8.22 g/L vs. 8.88 ± 7.35 g/L; mean difference, 0.39 g/L; 95% CI, −2.57 to 3.36; *p* = 0.95). Median preoperative serum creatinine levels were comparable between groups and no significant difference was observed in postoperative creatinine values (85 [60–114] µmol/L vs. 77 [66–100] µmol/L; *p* = 0.53). The median length of hospital stay was 1 [1–1] day in both the non-obese and obese groups (*p* = 0.627). Discharge on postoperative day 1 occurred in 89.8% and 86.3% of patients, respectively (absolute risk difference, −3.6 percentage points; 95% CI, −15.7 to 8.6; *p* = 0.768). Similarly, SFR did not differ significantly between obese and non-obese patients (94.1% vs. 89.8%; absolute risk difference, +4.3 percentage points; 95% CI, −5.8 to 14.3; *p* = 0.41). No statistically significant difference was observed in overall complication rates between the two groups (obese cohort: 5.88% vs. non-obese cohort: 6.78%; absolute risk difference, −0.9 percentage points; 95% CI, −10.0 to 8.2; *p* = 1.000). In post hoc exploratory analyses, after adjustment for cumulative stone diameter, obesity was not significantly associated with 30-day complications (OR = 0.69; 95% CI, 0.13–3.60; *p* = 0.664) or failure to achieve stone-free status (OR = 0.33; *p* = 0.174). Greater cumulative stone diameter was associated with higher odds of failure to achieve stone-free status (OR = 1.04 per mm; *p* = 0.042). In the non-obese cohort, one patient (1.69%) required readmission 2 weeks postoperatively due to severe bleeding; CT angiography identified an arteriovenous fistula, and selective angioembolization was successfully performed (Clavien–Dindo grade IIIb). One patient (1.69%) receiving therapeutic anticoagulation developed mild postoperative hematuria, which resolved with conservative management including intravenous fluids and diuretics (grade I). Another patient (1.69%) developed UTI symptoms on postoperative day one, requiring intravenous antibiotic therapy (grade II). Additionally, one patient (1.69%) was readmitted due to hematuria; CT angiography did not reveal an active bleeding source. Clot evacuation via the bladder catheter was performed (grade IIIa). In the obese group, one patient (1.96%) developed mild hematuria on postoperative day one, which was managed conservatively (grade I). One case (1.96%) of UTI was observed and treated with antibiotics (grade II). In addition, one patient (1.96%) with pre-existing anemia required blood transfusion (grade II) after the hemoglobin level decreased from 104 g/L preoperatively to 79 g/L on postoperative day 1. Each complication occurred in a different patient, and no patient experienced more than one postoperative complication. Detailed postoperative complications are summarized in Table 2.

## 4. Discussion

In the present study, no statistically significant differences were observed between the two groups in key postoperative parameters, including hemoglobin drop, SFR, postoperative day 1 discharge, or complication rates. Operative time was the only parameter that differed significantly between groups. However, obese patients also presented with significantly greater cumulative stone diameter. Importantly, after adjustment for cumulative stone diameter and age in multivariable regression analysis, obesity was no longer an independent predictor of operative time, whereas cumulative stone diameter remained significantly associated with prolonged operative duration. Consistent with this finding, BMI was not independently associated with operative time when analyzed as a continuous variable, while in an expanded post hoc sensitivity model incorporating additional stone-related and clinical covariates, obesity remained non-significantly associated with operative time and cumulative stone diameter remained a significant predictor. SSD was evaluated separately because of its close relationship with obesity and its potential role as a mediator of the technical effects of obesity. Inclusion of SSD in a sensitivity analysis did not materially alter the association between obesity and operative time, and no relevant multicollinearity was observed. In exploratory adjusted analyses, obesity was not significantly associated with either 30-day complications or failure to achieve stone-free status after adjustment for cumulative stone diameter, whereas greater stone burden was associated with higher odds of failure to achieve stone-free status. These findings suggest that differences in stone burden may account for a substantial part of the observed difference in operative time; however, residual confounding by stone complexity, anatomical characteristics, and other unmeasured factors cannot be excluded.

Although hypertension and diabetes mellitus were significantly more prevalent in the obese group, no statistically significant difference in the overall complication rate was observed between the groups [14,15]. A possible explanation is that all patients underwent comprehensive preoperative anesthesiology assessment, and comorbidities were medically optimized before surgery. Patients with uncontrolled medical conditions or untreated UTI were not scheduled for surgery until these issues had been adequately managed. In addition, our standardized perioperative infection protocol and the fact that all procedures were performed by a single experienced surgeon using a standardized technique may also have contributed to the low complication rates observed in both groups. Nevertheless, the relatively modest sample size and low overall complication rate may have limited the statistical power to detect small but clinically meaningful differences between the groups. Therefore, the absence of statistically significant differences should not be interpreted as evidence that obesity-related comorbidities have no effect on perioperative risk.

In developed countries, obesity represents a major public health problem driven by increased caloric intake and decreased physical activity. In obese patients, treatment options for stones may be more limited, as extracorporeal shock wave lithotripsy can be less feasible because of increased SSD and the maximum table weight limit [16]. Furthermore, according to Nussberger et al., a BMI >30 kg/m^2^ is associated with an increased risk of renal hematoma formation [17]. Notwithstanding its well-established efficacy, flexible ureteroscopy may require multiple treatment sessions in patients with high stone burden [18]. This is clinically relevant because obese patients frequently present with additional comorbidities, thereby increasing cumulative anesthetic and procedural risks associated with repeated interventions. When PCNL is selected as the treatment option, obesity introduces significant challenges for the urologist and anesthesiologist [19]. These challenges include compromised radiographic or ultrasonographic visualization, difficulty identifying anatomical landmarks, and limitations in reaching the kidney with conventional-length instruments and access sheaths due to excessive adipose tissue. In addition, safe patient positioning may be more demanding in this population. Failure to obtain optimal renal access, together with unfavorable pelvicalyceal angulation, and restricted instrument mobility may further negatively influence procedural outcomes [20]. Consequently, surgical expertise and meticulous preoperative planning are critical determinants of procedural success.

PCNL outcomes in patients with obesity remain heterogeneous across the available literature. The CROES study demonstrated a significant increase in operative time with increasing BMI [21]. In patients with BMI > 40 kg/m^2^, operative times were prolonged by almost 30 min compared with those of normal-weight patients. Furthermore, increasing BMI was associated with lower SFR and higher reintervention rates, potentially reflecting greater technical challenges and the higher prevalence of staghorn stones in this population. Notably, length of hospital stay did not differ significantly between obese and normal-weight patients. In contrast, Torrecilla Ortiz et al. found that obesity did not significantly influence SFR or complication rates after PCNL [22]. Although operative time tended to increase with BMI, the difference was not statistically significant. However, radiation exposure increased significantly with higher BMI. Cormio et al. analyzed outcomes of mini and standard PCNL in obese patients in a large single-surgeon series [9]. They found that obese and non-obese patients had similar surgical outcomes, with no significant differences in operative time, SFR, complication rate, or hospital stay. Within the obese population, mini-PCNL was associated with shorter operative time, higher SFR, and lower blood transfusion rates than standard PCNL. Similarly, Slade et al. reported that mini-PCNL provides comparable safety and efficacy in obese and non-obese patients [23].

A recent study by Gelmis et al. specifically evaluated obese patients undergoing mini-PCNL in the supine or prone position, representing one of the closest comparable cohorts to the present study [8]. In their analysis, supine mini-PCNL was associated with reduced fluoroscopy time, shorter hospital stay, and fewer major complications compared with prone mini-PCNL. Similarly, in the present study, obese patients undergoing supine mini-PCNL demonstrated favorable perioperative outcomes and a low complication profile, further supporting the safety and feasibility of this approach. In addition, their study compared supine and prone mini-PCNL exclusively within an obese population, whereas our analysis focused on the impact of obesity itself within a standardized supine mini-PCNL cohort. Overall, these findings suggest that obesity alone may not necessarily preclude consideration of mini-PCNL in appropriately selected patients. From a clinical perspective, these findings suggest that surgical decision-making should not be based on BMI alone. Obesity-related factors, including increased SSD, greater stone burden, and associated comorbidities, should be considered individually during preoperative assessment and patient counseling. Further prospective studies are needed to determine whether specific obesity-related factors should influence patient selection, perioperative management, or postoperative follow-up.

Importantly, BMI alone may not be the most relevant predictor of PCNL outcomes. Cakmak et al. evaluated the impact of different abdominal fat parameters and found that BMI, as well as visceral and subcutaneous fat areas, were not significantly associated with PCNL success [24]. Instead, abdominal circumference measured on CT and stone surface area were identified as independent predictors of PCNL success. These findings suggest that anthropometric parameters reflecting abdominal fat distribution may better capture anatomical factors influencing procedural difficulty.

The optimal patient positioning during PCNL (prone versus supine) remains controversial. Several theoretical and previously reported advantages of the supine position have been described, particularly in obese patients, including improved airway control for the anesthesiologist, reduced radiation exposure for the surgeon, lower intrarenal pressure, facilitation of spontaneous fragment clearance, and elimination of patient repositioning and redraping during the procedure [25]. These advantages may be particularly relevant in obese patients, where turning the patient into the prone position can be technically demanding and may require additional personnel. However, as the present study did not include a prone comparator group, no conclusions regarding positional superiority can be drawn.

Regarding SFR assessment, the use of X-ray and ultrasound may have resulted in overestimation of true SFR, given the superior sensitivity of non-contrast CT in detecting residual fragments [26,27]. In addition, residual fragments <4 mm were considered clinically insignificant, whereas contemporary EAU guideline definitions apply stricter thresholds. Therefore, the reported SFRs should be interpreted cautiously when compared with studies using routine non-contrast CT follow-up and stricter stone-free definitions. Although the same imaging protocol and SFR criteria were applied in both groups, potential BMI-related differences in imaging sensitivity should be considered when interpreting between-group comparisons.

This study has several limitations. First, it is a retrospective single-center analysis, which may limit generalizability and introduce selection bias. Although all consecutive patients meeting the predefined eligibility criteria were included, the study population consisted exclusively of patients undergoing supine mini-PCNL at our institution and therefore reflects the patient population and surgical practice of a single center. Consequently, the findings may not be directly generalizable to patients undergoing other PCNL techniques or to different clinical settings. Although multivariable regression analysis was performed to adjust for measured baseline differences in cumulative stone diameter and age, residual confounding and potential selection bias inherent to the retrospective study design could not be completely excluded. In addition, cumulative stone diameter was used as a surrogate measure of stone burden. This approach may not fully reflect total stone burden, particularly in patients with multiple stones or staghorn calculi. Volumetric or surface area-based stone burden assessment was not performed in the present study, which should be considered a limitation. Fluoroscopy time/radiation exposure and access/puncture time were not prospectively recorded in a standardized manner and could not be reliably reconstructed retrospectively. Furthermore, all procedures were performed by a single surgeon. Although this reduced variability in surgical technique, it may limit the applicability of the results to other centers and surgeons with different levels of experience. However, the operating surgeon had substantial prior experience in PCNL and had surpassed the initial learning curve before study initiation. In addition, the sample size was relatively limited, and multiple secondary outcomes were assessed without formal adjustment for multiplicity, increasing the risk of type I error. Therefore, secondary outcome analyses should be interpreted as exploratory and hypothesis-generating rather than confirmatory. Moreover, the relatively limited sample size and the low absolute complication rate may have reduced the statistical power to detect clinically meaningful differences between groups, particularly for the primary endpoint. The low number of outcome events also precluded reliable multivariable adjustment for all potentially relevant confounders; therefore, the logistic regression analyses were limited to parsimonious exploratory models. The wide confidence intervals around the effect estimates indicate substantial uncertainty and include potentially clinically meaningful differences in either direction. Consequently, the absence of statistically significant differences should not be interpreted as evidence of equivalence, rather it indicates that no significant difference in complication rates was observed in this cohort. Furthermore, SFR was assessed using KUB radiography and ultrasonography rather than routine non-contrast CT. As these imaging modalities have limited sensitivity for detecting residual fragments, the true SFR may have been overestimated. Moreover, their diagnostic performance may be affected by body habitus, potentially introducing differential detection bias between the BMI groups. This is particularly relevant given the numerically higher SFR observed in the obese group (94.1% vs. 89.8%), as residual fragments may have been less readily detected in patients with higher BMI. Therefore, the absence of a statistically significant difference in SFR should be considered exploratory and should not be interpreted as evidence of equivalence between obese and non-obese patients. Furthermore, as our institution is a tertiary referral center, many patients were referred back to their originating institutions after postoperative follow-up; therefore, subsequent management of residual fragments, including observation or auxiliary procedures, could not be systematically captured. Finally, because the present study did not include a comparator group undergoing prone mini-PCNL or standard PCNL, our findings should not be interpreted as demonstrating superiority of supine mini-PCNL over other PCNL techniques or patient positions. Instead, these findings support the feasibility of standardized supine mini-PCNL within the present cohort and should not be interpreted as a broad recommendation for clinical practice. Larger prospective multicenter studies are warranted to validate these findings and determine their generalizability.

## 5. Conclusions

In this retrospective single-center cohort, no statistically significant differences were observed between obese and non-obese patients regarding hemoglobin drop, SFR, early discharge, or complication rates following supine mini-PCNL. Although operative time was longer in obese patients, this difference was no longer significant after adjustment for stone size and age, suggesting that differences in stone burden may have contributed substantially to the observed difference in operative time. Given the retrospective design, relatively small sample size, low number of complication events, and non-CT-based assessment of SFR, these findings should be interpreted with caution. Rather than demonstrating equivalence, our results indicate that no significant differences were observed within this cohort. These findings support the feasibility of standardized supine mini-PCNL in obese patients within our cohort but should not be interpreted as a broad recommendation for clinical practice. Larger prospective, multicenter studies are warranted to validate these findings, determine their generalizability, and further define the impact of obesity-related anatomical factors on surgical complexity and outcomes.

## Figures and Tables

**Table 1:** Demographic and clinical characteristics of the study population.

Variables	Non-Obese (BMI < 30 kg/m^2^) Group	Obese (BMI ≥ 30 kg/m^2^) Group	*p*-Value
Patients (*n*, %)	59 (53.64)	51 (46.36)	
Age (years), median [IQR]	53 [36–66]	58 [46–63]	0.181
Sex			
male [No. (%)]	27 (45.76)	29 (56.86)	0.246
female [No. (%)]	32 (54.24)	22 (43.14)	
BMI (kg/m^2^), mean ± SD	25.48 ± 3.20	35.07 ± 3.62	<0.001
ASA score 1 [No. (%)]	10 (16.95)	2 (3.92)	0.055
ASA score 2 [No. (%)]	41 (69.49)	37 (72.55)	
ASA score 3 [No. (%)]	8 (13.56)	12 (23.53)	
Hypertension [No. (%)]	20 (33.90)	31 (60.78)	0.005
Diabetes mellitus [No. (%)]	12 (20.34)	21 (41.18)	0.017
Laterality			
left side [No. (%)]	36 (61.02)	33 (64.73)	0.690
right side [No. (%)]	23 (38.98)	18 (35.29)	
Guy’s stone score 1 [No. (%)]	28 (47.46)	21 (41.18)	0.400
Guy’s stone score 2 [No. (%)]	27 (45.76)	22 (43.14)	
Guy’s stone score 3 [No. (%)]	2 (3.39)	6 (11.76)	
Guy’s stone score 4 [No. (%)]	2 (3.39)	2 (3.92)	
Cumulative stone diameter (mm), median [IQR]	21 [16–26]	30 [20–43]	<0.001
Stone density (HU), mean ± SD	1046.83 ± 365.02	869.69 ± 311.67	0.008
SSD (cm), median [IQR]	8.78 [7.97–9.60]	12.61 [11.24–14.03]	<0.001
Stone location			
lower calyx [No. (%)]	5 (8.47)	2 (3.92)	0.626
middle calyx [No. (%)]	1 (1.69)	3 (5.88)	
upper calyx [No. (%)]	3 (5.08)	2 (3.92)	
renal pelvis [No. (%)]	19 (32.20)	15 (29.41)	
partial staghorn [No. (%)]	1 (1.69)	4 (7.84)	
complete staghorn [No. (%)]	2 (3.39)	2 (3.92)	
multiple stones [No. (%)]	28 (47.46)	23 (45.10)	
Number of access tracts			
single tract [No. (%)]	56 (94.92)	49 (96.08)	1
multitract (2 tracts) [No. (%)]	3 (5.08)	2 (3.92)	
Puncture site			
lower calyx [No. (%)]	43 (72.88)	31 (60.78)	0.378
middle calyx [No. (%)]	8 (13.56)	11 (21.57)	
upper calyx [No. (%)]	5 (8.47)	7 (13.73)	
lower and middle calyx [No. (%)]	1 (1.69)	2 (3.92)	
lower and upper calyx [No. (%)]	2 (3.39)	0 (0.00)	
Preoperative urine culture positivity [No. (%)]	20 (33.90)	16 (31.37)	0.778
Preoperative creatinine (µmol/L), median [IQR]	75 [64–93]	81 [64–103]	0.400
Preoperative Hgb (g/L), mean ± SD	136.95 ± 14.82	144.55 ± 16.23	0.012

ASA, American Society of Anesthesiologists; BMI, body mass index; Hgb, hemoglobin; HU, Hounsfield unit; IQR, interquartile range; SD, standard deviation; SSD, skin-to-stone distance.

**Table 2:** Postoperative complications according to the modified Clavien–Dindo classification.

Complication	Clavien–Dindo Grade	Timing	Management	Non-Obese (*n*, %)	Obese (*n*, %)	*p*-Value
**Overall complications**	I–IIIb	–	According to complication	4 (6.78%)	3 (5.88%)	1.000
**Minor complications**	I–II	–	Conservative/medical treatment	3 (5.08%)	3 (5.88%)	0.661
Hematuria	I	POD 1	Intravenous fluids and diuretics	1 (1.69%)	1 (1.96%)	1.000
UTI	II	POD 1	Intravenous antibiotics	1 (1.69%)	1 (1.96%)	1.000
Blood transfusion	II	POD 1	Blood transfusion	0 (0%)	1 (1.96%)	0.464
**Major complications**	IIIa–IIIb	–	Interventional management	2 (3.39%)	0 (0%)	0.498
Hematuria requiring clot evacuation	IIIa	7 days after surgery	CT angiography, clot evacuation	1 (1.69%)	0 (0%)	1.000
Arteriovenous fistula	IIIb	14 days after surgery	CT angiography, selective angioembolization	1 (1.69%)	0 (0%)	1.000
**Readmission**	IIIa–IIIb	within 14 days	Interventional management	2 (3.39%)	0 (0%)	1.000

CT, computed tomography; POD, postoperative day; UTI, urinary tract infection.

## Data Availability

The data supporting the findings of this study are available from the corresponding author upon reasonable request.

## Abbreviations

The following abbreviations are used in this manuscript:

ASA	American Society of Anesthesiologists
BMI	Body mass index
CT	Computed tomography
DJ stent	Double J stent
IQR	Interquartile range
KUB radiography	Kidney–ureter–bladder radiography
PCNL	percutaneous nephrolithotomy
mini-PCNL	mini-percutaneous nephrolithotomy
SD	standard deviation
SFR	stone-free rate
SSD	skin-to-stone distance
UTI	urinary tract infection
